# Long-term use of selective digestive decontamination in an ICU highly endemic for bacterial resistance

**DOI:** 10.1186/s13054-018-2057-2

**Published:** 2018-05-30

**Authors:** Catalina Sánchez-Ramírez, Silvia Hípola-Escalada, Miriam Cabrera-Santana, María Adela Hernández-Viera, Liliana Caipe-Balcázar, Pedro Saavedra, Fernando Artiles-Campelo, Nayra Sangil-Monroy, Carlos Federico Lübbe-Vázquez, Sergio Ruiz-Santana

**Affiliations:** 1 Intensive Care Unit, Hospital Universitario de Gran Canaria Dr. Negrín, Las Palmas de Gran Canaria, La Ballena s/n, E-35010 Las Palmas, Spain; 2 Mathematics Department, Universidad de las Palmas de Gran Canaria, Las Palmas, Spain; 3 Microbiology Department, Hospital Universitario de Gran Canaria Dr. Negrín, Las Palmas de Gran Canaria, Las Palmas, Spain; 4 Pharmacy Department, Hospital Universitario de Gran Canaria Dr. Negrín, Las Palmas de Gran Canaria, Las Palmas, Spain

**Keywords:** Selective digestive decontamination, Drug resistance, ICU-acquired infection, Ventilator-associated pneumonia, Multidrug-resistant pathogens, Bloodstream infection, Colistin, Tobramycin

## Abstract

**Background:**

We examined whether long-term use of selective digestive tract decontamination (SDD) was effective in reducing intensive care unit (ICU)-acquired infection and antibiotic consumption while decreasing colistin-, tobramycin-, and most of the antibiotic-resistant colonization rates in a mixed ICU with a high endemic level of multidrug-resistant bacteria (MDRB).

**Methods:**

In this cohort study, which was conducted in a 30-bed medical-surgical ICU, clinical outcomes before (1 year, non-SDD group) and after (4 years) implementation of SDD were compared. ICU patients who were expected to require tracheal intubation for > 48 hours were given a standard prophylactic SDD regimen. Oropharyngeal and rectal swabs were obtained on admission and once weekly thereafter.

**Results:**

ICU-acquired infections occurred in 110 patients in the non-SDD group and in 258 in the SDD group. A significant (*P* <  0.001) reduction of infections caused by MDRB (risk ratio [RR], 0.31; 95% CI, 0.23–0.41) was found after SDD and was associated with low rates of colistin- and tobramycin-resistant colonization. Colistin- and tobramycin-acquired increasing rate of ICU colonization resistance by 1000 days, adjusted by the rate of resistances at admission, was nonsignificant (0.82; 95% CI, 0.56 to 1.95; 1.13; 95% CI, 0.75 to 1.70, respectively). SDD was also a protective factor for ICU-acquired infections caused by MDR gram-negative pathogens and *Acinetobacter baumannii* in the multivariate analysis. In addition, a significant (*P* <  0.001) reduction of ventilator-associated pneumonia (VAP) (RR, 0.43; 95% CI, 0.32–0.59) and secondary bloodstream infection (BSI) (RR, 0.35; 95% CI, 0.24–0.52) was found. A decrease in antibiotic consumption was also observed.

**Conclusions:**

Treatment with SDD during 4 years was effective in an ICU setting with a high level of resistance, with clinically relevant reductions of infections caused by MDRB, and with low rates of colistin- and tobramycin-resistant colonization with nonsignificant increasing rate of ICU colonization resistance by 1000 days, adjusted by the rate of resistances at ICU admission. In addition, VAP and secondary BSI rates were significantly lower after SDD. Notably, a decrease in antimicrobial consumption was also observed.

## Background

Selective digestive decontamination (SDD) is a prophylactic treatment for critically ill patients that is based on an oropharyngeal paste and enteral suspension containing antimicrobials, usually tobramycin, colistin, and an antifungal as well as an intravenous antibiotic, administered during the first 4 days of intensive care unit (ICU) admission (usually a second-generation cephalosporin). The aim of SDD is to prevent or eradicate, if present, the oropharyngeal and intestinal abnormal carriage of potentially pathogenic microorganisms, such as aerobic gram-negative bacilli (AGNB), methicillin-sensitive *Staphylococcus aureus*, and yeasts, in patients at risk for nosocomial infections [1, 2]. Once a patient has been successfully decolonized, the unaffected anaerobic flora would offer prevention against new colonization with potential pathogenic microorganisms. In critically ill patients, SDD has been proven to prevent severe infections [1–3] and to reduce mortality [3, 4], particularly in settings with a low prevalence of multidrug-resistant bacteria. However, the use of SDD is still a matter of debate, largely because of concerns that it may promote the emergence of antibiotic-resistant strains [5, 6]. Also, the effect of SDD in ICUs with endemic circulation of multidrug-resistant gram-negative bacilli MDR-GNB) remains controversial [7, 8]. We investigated whether long-term use of SDD was efficacious in reducing ICU-acquired MDR-GNB infection and also sought to determine its effect, including colistin- and tobramycin-resistant colonization as well as other nosocomial infections and subsequent antibiotic consumption, in a mixed ICU with a high endemic level of multidrug-resistant bacteria (MDRB).

## Methods

### Study design and patients

We conducted a prospective cohort study in a 30-bed medical-surgical ICU of an acute care tertiary hospital in Las Palmas de Gran Canaria, Canary Islands, Spain. All consecutive patients admitted to the ICU between September 1, 2010, and September 30, 2015, were included. They were grouped into two consecutive cohorts before and after implementation of SDD. Data of both cohorts were collected prospectively. Patients admitted between September 1, 2010, and September 30, 2011, were included in the non-SDD cohort, and patients admitted between October 1, 2011, and September 30, 2015, were included in the SDD cohort. Since October 1, 2011, SDD measures have been systematically applied to all ICU patients expected to require tracheal intubation for more than 48 hours (SDD cohort). SDD was started when the “Pneumonia Zero” project began to be implemented among Spanish ICUs. In the “Pneumonia Zero” project, SDD was a highly recommended component of the ventilator-associated pneumonia (VAP) prevention bundle [9]. The primary objective was to compare outcome measures between the non-SDD and SDD cohorts.

### SDD protocol

SDD was started on the day of tracheal intubation and was given throughout the length of the ICU stay and until discharge from the ICU. Patients were treated three times daily with 1 g of an oral paste applied to the oral cavity. The composition per 1 g was 20 mg of 2% colistin, 30 mg of 3% tobramycin, and 20 mg of 2% nystatin. The patients also received a 14-ml suspension containing 140 mg of 1% colistin, 180 mg of 2% tobramycin, and 453.6 mg of 3.2% nystatin [10], which was administered into the gut through a nasogastric tube. In tracheostomized patients, the oral paste was also applied on the skin surrounding the tracheostomy three times daily. Enteral vancomycin, 40 mg of 4% oropharyngeal paste, and 700 mg of vancomycin in digestive solution were added at the same 8-hour interval to all methicillin-resistant *Staphylococcus aureus* (MRSA) carriers, as well as to patients referred from elsewhere until MRSA noncarrier status was documented [11]. All patients received systemic cefotaxime, 1 g every 8 hours, during the first 4 days of SDD therapy, except patients with infections on admission, who were treated with their antibiotics.

### Endpoints

The primary endpoints of the study were the incidence of ICU-acquired infection caused by MDRB, the evolution of colistin- and tobramycin-resistant colonization, and the clinical impact of SDD on MDRB infections. Secondary endpoints were VAP, central line-associated primary bloodstream infection (CLABSI), secondary bloodstream infection (BSI), urinary tract infection, and antibiotic consumption.

### Study procedures and definitions

Surveillance samples from the throat, rectum, tracheostomy, and pressure sores were collected on ICU admission and once weekly thereafter. Diagnostic samples from tracheal aspirates, peripheral blood, urine, or wounds were obtained at the physician’s discretion. Antimicrobial susceptibility testing was performed with the VITEK-2 system (bioMérieux, Inc., Durham, NC, USA) [12], with breakpoints defined according to the Clinical and Laboratory Standards Institute [13] and the European Committee on Antimicrobial Susceptibility Testing [14] guidelines. Infections caused by MDRB included the following:

1. *Enterobacteriaceae* spp. resistant to ceftazidime and/or aminoglycosides and/or ciprofloxacin with extended-spectrum β-lactamase (ESBL) producing bacteria
2. *Pseudomonas aeruginosa* resistant to ceftazidime and/or aminoglycosides and/or ciprofloxacin and/or imipenem
3. MRSA
4. Any strain of *Acinetobacter* spp. resistant to carbapenems
5. Gram-negative bacteria resistant to three or more antimicrobial families
6. *Clostridium difficile*
7. Vancomycin-resistant *Enterococcus* spp.

Imported MDRB infection was considered when cultures of surveillance or diagnostic samples were positive within 48 hours of ICU admission. ICU-acquired MDRB infection was defined as isolation of a new strain that was not recovered in any of the samples taken during the first 48 hours of admission. Also, secondary endogenous infections were those preceded by gastrointestinal carriage of MDRB with identical antibiotic susceptibility patterns and exogenous infections when the infecting MDRB was isolated in diagnostic samples without previous colonization [15].

ICU-acquired infections were collected from the ENVIN-HELICS registry (National Nosocomial Infection Surveillance Study–Hospitals in Europe Link for Infection Control through Surveillance), which is a nationwide ongoing multicenter data collection system designed to record invasive device-related infections in ICU patients (http://hws.vhebron.net/envin-helics/). Diagnostic criteria established by the ENVIN-HELICS project were used [16]. The diagnosis of VAP included the following:

1. Sequential chest x-rays or computed tomographic (CT) scans with an image suggestive of pneumonia (two or more radiographs or CT scans in the presence of underlying cardiac or pulmonary disease)

2. Fever (> 38 °C) and/or leukocytosis (≥ 12,000 white blood cells [WBC]/mm^3^) or leukopenia (≤ 4000 WBC/mm^3^)

3. At least one of the following:

a. New-onset purulent sputum or change in the characteristics of sputum

b. Cough, dyspnea, or tachycardia

c. Rales or bronchial breath sounds on auscultation, ronchi, wheezing

d. Worsening gas exchange

Other infections were diagnosed according to the Centers for Disease Control and Prevention definitions [17] when applicable to ICU patients.

### Ethics

Our ICU participated in the ENVIN-HELICS national registry, and we used this registry for prospective data collection during the study [18]. Baseline data collection started in 2010. The ENVIN-HELICS registry was approved by the ethics committees of the majority of participating ICUs and was declared a registry of healthcare interest by the Spanish Ministry of Health, Social Services and Equality in 2014. The ENVIN-HELICS registry was also approved by our hospital’s ethics committee. We applied SDD in the context of the Spanish national “Pneumonia Zero” project [9], the framework for implementing SDD, which is supported by the Spanish Ministry of Health, Social Policy and Equality through a contract with the Spanish Society of Critical Care Medicine and Coronary Units (number 0100/2010/0784). The study protocol was approved by the Clinical Research Ethics Committee of Hospital del Mar (Barcelona, Spain), which was the national reference committee.

### Statistical analysis

Categorical variables are expressed as frequencies and percentages, and quantitative variables are expressed as mean ± SD or median and IQR (25th–75th percentiles) as appropriate. Percentages were compared with the χ^2^ test, means with Student’s *t* test, and medians with the Wilcoxon test for independent data. Statistically significant variables in the univariate analysis were introduced in a multivariate logistic regression model, with selection of variables based on a complete enumeration algorithm and the Bayes information criterion. The models were summarized as coefficients (β), SE, *P* values (likelihood ratio test), and ORs, which were estimated by 95% CIs.

For each ICU-acquired infection, the incidence per 1000 days of exposure in each cohort and the corresponding relative risks (RRs) were obtained by Poisson regression analysis. Specifically, for the *i*th cohort determined by hospital, year, and month, we denote by *m*_*i*_ the number of events and by *d*_*i*_ the number of days of exposition (for all patients). A random effects Poisson model [19] was considered, which assumes that, *m*_*i*_~*Poisson*(*υ*_*i*_*μ*_*i*_) is:

$$ \log {\mu}_i=\log {d}_i+\alpha +\beta \bullet {SDD}_i $$

where *υ*_1_, …, *υ*_*k*_ are continuous positive valued *i*dd random variables such that *E*[*υ*_*i*_] = 1 and var(*υ*_*i*_) = *τ*. *SDD* is 1/0 according presence/absence of SDD. The parameter *τ* is the overdispersion. The RR deduced from the model is *RR* = exp *β*. The model was estimated by the likelihood method and summarized by the RRs, which were estimated by 95% CIs. Statistical significance was set at *P* ≤ 0.05. Data were analyzed using the R package, version 3.3.1 (R Development Core Team, 2016) [20].

## Results

**Fig. 1:**
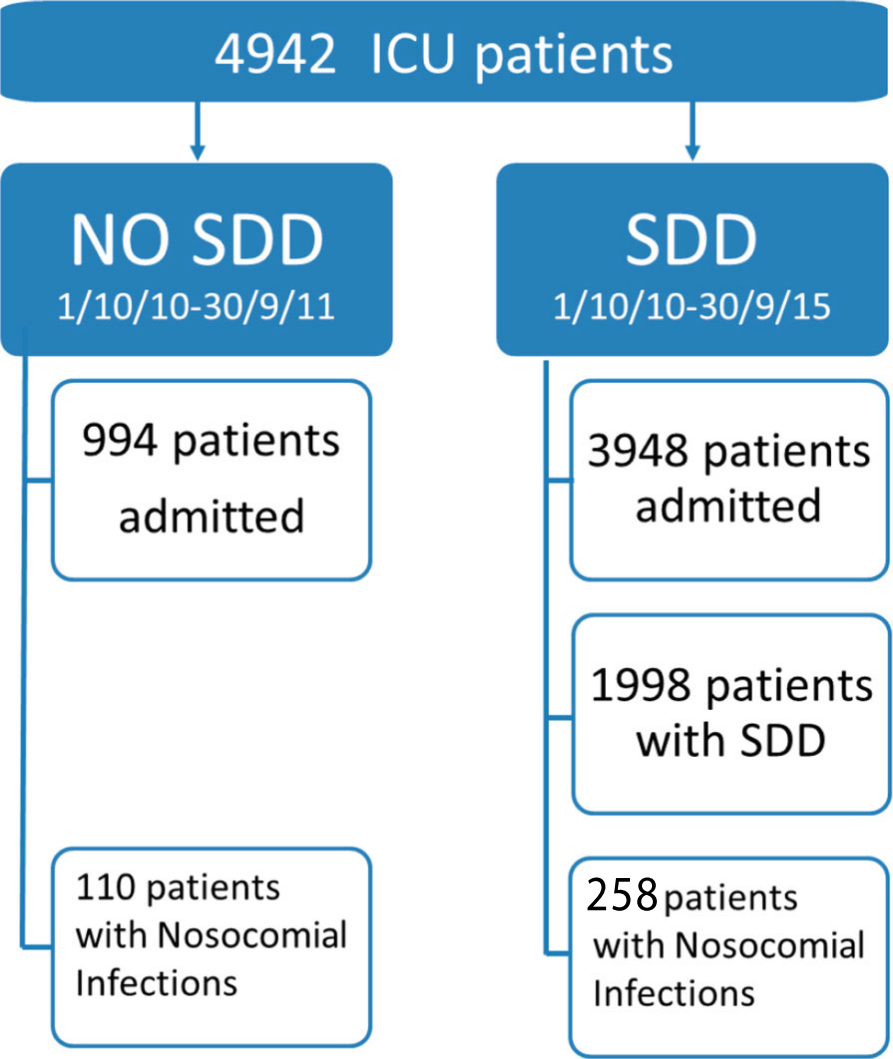
Patient flowchart. *SDD* Selective digestive tract decontamination

During the 5-year study period, 3948 critically ill patients were admitted to the ICU, and ICU-acquired infection (VAP, CLABSI, secondary BSI, urinary tract infection) was diagnosed in 368 of them (7.8%). Of a total of 994 patients admitted to the ICU between September 2010 and September 2011, 110 patients had ICU-acquired infection in the non-SDD cohort. Of the 3948 patients admitted between October 2011 and September 2015, SDD was administered to 1998 (50.6%), and 258 developed an ICU-acquired infection (SDD cohort) (Fig. 1). No complications related to the use of SDD were recorded.

Results of univariate analysis are shown in Table 1. Demographic data and the distribution of most variables were similar in both cohorts. In the non-SDD cohort, the percentage of patients with chronic obstructive pulmonary disease and CLABSI was significantly lower than in the SDD cohort. However, we observed significantly lower rates of infections caused by MRDB, including *Acinetobacter* spp., other GNB and ESBL-producing multidrug-resistant bacteria, VAP, and secondary BSI, in the SDD cohort than in the non-SDD cohort. A significantly higher number of patients with CLABSI in the SDD cohort than in the non-SDD cohort was found. ICU-acquired infections caused by *C. difficile* or vancomycin-resistant *Enterococcus* spp. did not occur. In the multivariate analysis, SDD was found to be a protective factor against ICU-acquired infections caused by *Acinetobacter* spp. and MDR-GNB (Table 2). In the multivariate logistic regression model for MDRB infection, renal replacement therapy (OR, 2.130; 95% CI, 1.346–3.372; *P* = 0.001) was an independent risk factor for MDRB infection, whereas SDD was a protective factor (OR, 0.491; 95% CI, 0.305–0.790; *P* <  0.001).

**Table 1:** Results of univariate analysis

Variables	Non-SDD cohort (*n* = 110)	SDD cohort (*n* = 258)	*P* value
Male sex	74 (67.3)	166 (64.3)	0.589
Age, years, mean ± SD	59.5 ± 15.8	60.7 ± 16.4	0.539
APACHE II score on admission, mean ± SD	21.2 ± 7.7	22.0 ± 7.7	0.345
Glasgow Coma Scale score, median (IQR)	15 (8–15)	14.5 (8–15)	0.098
Diagnosis on ICU admission			0.289
Medical	79 (71.8)	190 (73.6)	
Scheduled surgery	10 (9.1)	33 (12.8)	
Emergency surgery	21 (19.1)	35 (13.6)	
Septic response			0.399
Sepsis	57 (52.8)	110 (45.45)	
Septic shock	51 (47.2)	132 (54.55)	
Prior surgery	18 (16.4)	37 (14.3)	0.618
Urgent surgery	34 (30.9)	70 (27.1)	0.461
Trauma patients	17 (15.5)	31 (12.0)	0.370
Current smokers	21 (19.1)	31 (27.4)	0.141
Underlying illness			
Diabetes mellitus	34 (30.9)	86 (33.3)	0.650
Coronary artery disease	19 (17.3)	45 (17.4)	0.969
Chronic liver disease	6 (5.5)	18 (7.0)	0.588
Chronic obstructive lung disease	9 (8.2)	43 (16.7)	0.032
Solid neoplasm	10 (9.1)	26 (10.1)	0.771
Chronic renal failure	40 (36.4)	56 (21.7)	0.003
Renal replacement therapy	34 (30.9)	91 (35.3)	0.419
Parenteral nutrition	26 (23.6)	50 (19.4)	0.356
Immunosuppression	8 (7.3)	22 (8.5)	0.687
Malnutrition	12 (10.9)	24 (9.3)	0.635
ICU-acquired infection			
VAP	59 (53.6)	102 (39.5)	0.013
CLABSI	26 (23.6)	106 (41.1)	0.001
Secondary BSI	31 (28.2)	47 (18.2)	0.023
Urinary tract infection	29 (26.4)	73 (28.3)	0.705
Infections caused by MDRB			
Gram-negative bacilli	12 (10.9)	8 (3.1)	0.002
*Acinetobacter* spp.	13 (11.8)	3 (1.2)	< 0.001
ESBL-producing MDRB	38 (34.5)	62 (24.0)	0.038
*Pseudomonas aeruginosa*	10 (9.1)	23 (8.9)	0.957
Methicillin-resistant *Staphylococcus aureus*	4 (3.6)	5 (1.9)	0.460
ICU stay, days, median (IQR)	28 (16–45)	33 (17–50)	0.192
ICU mortality	36 (32.7)	85 (33.2)	0.929

*Abbreviations: SDD* Selective digestive tract decontamination, *ICU* Intensive care unit, *APACHE* Acute Physiology and Chronic Health Evaluation, *VAP* Ventilator-associated pneumonia, *CLABSI* Central line-associated bloodstream infection, *BSI* Bloodstream infection, *MDRB* Multidrug-resistant bacteria, *ESBL* Extended-spectrum β-lactamase

Data are expressed as frequency and percent unless otherwise stated

**Table 2:** Results of multivariate logistic regression analysis for selective digestive tract decontamination

Variable	*P* value	OR (95% CI)
CLABSI	0.003	2.218 (1.307 to 3.764)
*Acinetobacter* spp.	< 0.001	0.091 (0.025 to 0.329)
MDR-GNB	0.001	0.204 (0.079 to 0.527)

*CLABSI* Central line-associated bloodstream infection, *MDR-GNB* Multidrug-resistant gram-negative bacilli

Treatment with SDD was associated with a significant reduction of the RR for ICU-acquired infections caused by MDRB, VAP, and secondary BSI (Table 3). The probabilities of acquiring infections caused by MDRB, VAP, and secondary BSI were 69%, 57%, and 66% lower, respectively, in the SDD cohort than in the non-SDD cohort.

**Table 3:** Intensive care unit-acquired infection rates

	Non-SDD cohort (*n* = 110)	SDD cohort (*n* = 258)	*P* value	Risk ratio (95% CI)
VAP/MV days				
Number of VAP	63	110	< 0.001	0.437 (0.320 to 0.595)
Days of MV	6112	24,432		
VAP/1000 MV days	10.3	4.5		
Urinary tract infection/urinary catheter days				
Number of urinary tract infections	33	97	0.110	0.725 (0.488 to 1.076)
Days of indwelling urinary catheter	8707	35,312		
Urinary infections/1000 catheter days	3.79	2.75		
CLABSI/CVC days				
Number of CLABSI			0.802	1.056 (0.690 to 1.615)
Days of CVC	7249	30,631		
CLABSI/1000 CVC days	3.59	3.9		
Secondary BSI/ICU days				
Number of secondary BSI	43	57	< 0.001	0.349 (0.237 to 0.516)
ICU days of stay	9176	37,857		
Secondary BSI/1000 ICU days	4.69	1.64		
MDRB/ICU days				
Number of MDRB infections	88	112	< 0.001	0.308 (0.233 to 0.408)
ICU days of stay	9176	37,857		
MDRB infections/1000 ICU days	9.59	2.96		

*Abbreviations: SDD* Selective digestive tract decontamination, *VAP* Ventilator-associated pneumonia, *MV* Mechanical ventilation, *CLABSI* Central line-associated bloodstream infection, *CVC* Central venous catheter, *BSI* Bloodstream infection, *MDRB* Multidrug-resistant bacteria

The consumption of nine antimicrobial agents commonly used in critically ill patients for treating MDRB, expressed as defined daily dose per 100 bed-days in the ICU, also showed a marked reduction after implementation of the SDD prophylactic strategy (Table 4). During the study period, other maneuvers directed toward reducing the use of antimicrobials were not applied.

**Table 4:** Antibiotic consumption during the study period

Drug	Non-SDD period (1 year)	SDD period (4 years)			
		1st year	2nd year	3rd year	4th year
Levofloxacin	59.01	38.10	50.79	43.96	13.89
Meropenem	43.09	32.46	32.30	27.9	11.10
Imipenem	25.08	10.20	12.57	6.06	3.15
Colistin	19.17	10.78	12.13	4.98	0.43
Vancomycin	7.23	4.95	6.96	6.56	2.47
Tobramycin	10.32	3.69	1.89	1.87	0.55
Amikacin	3.13	4.28	3.10	3.08	2.47
Ceftazidime	7.29	5.48	5.12	10.93	5.80
Ciprofloxacin	9.61	12.85	8.50	8.62	8.45
Cefotaxime	6.01	22.6	22.3	22.7	22.7

*SDD* Selective digestive tract decontamination

Data are expressed as defined daily dose per 100 bed-days in the intensive care unit

**Fig. 2:**
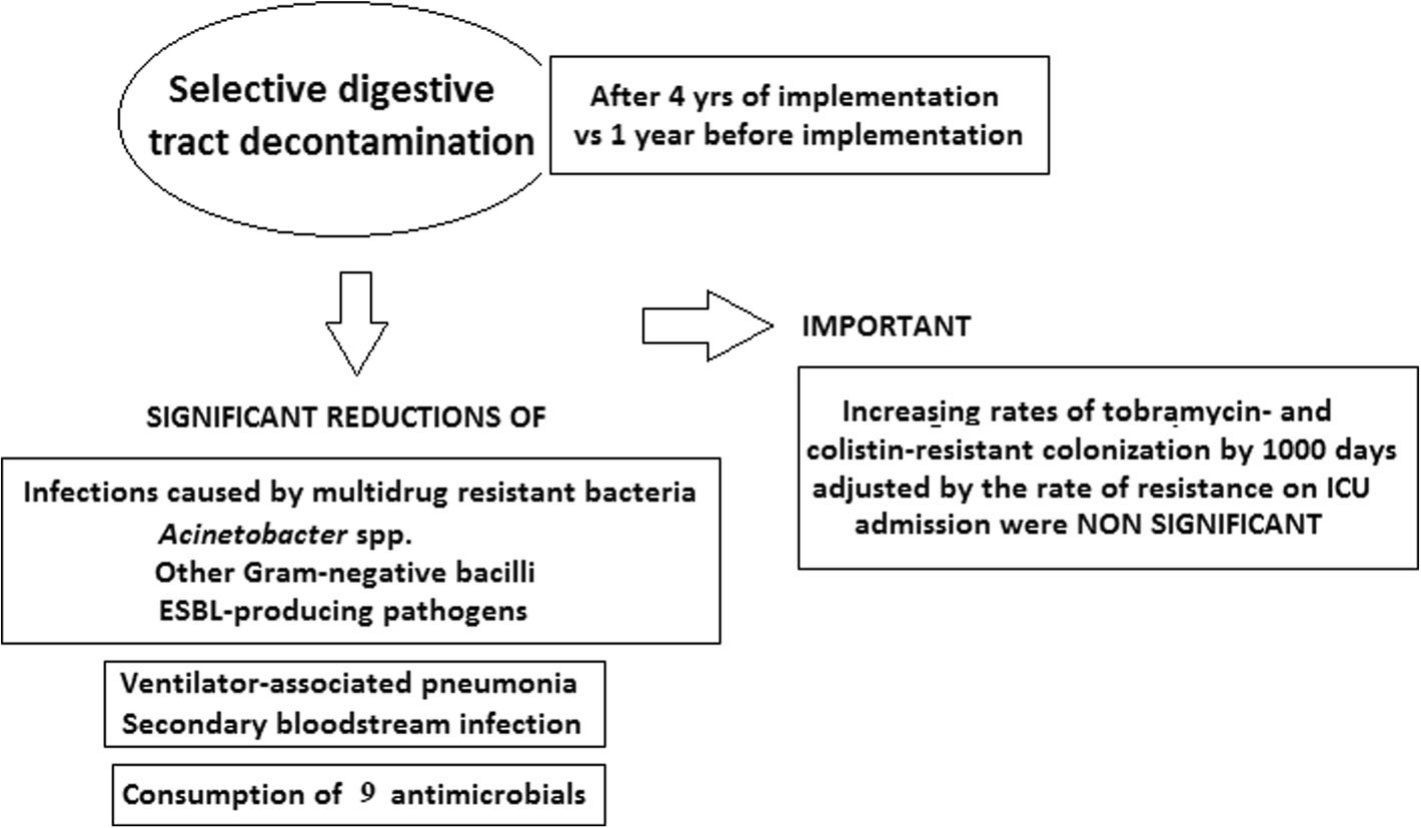
Summary of study findings. *ESBL* Extended-spectrum β-lactamase

Of a total of 3948 patients admitted to the ICU during the 4-year period of implementation of the SDD treatment, 285 showed surveillance samples colonized by colistin- or tobramycin-resistant pathogens. As shown in Table 5, there were increases of colonization resistance to colistin and tobramycin at ICU admission. Also, as shown in Table 5, the estimated rates adjusted to 100 patients with SDD decreased in the fourth year for tobramycin-resistant colonization and increased from 1.6 to 1.8 for colistin-resistant colonization in the third and fourth years of the study. The colistin- and tobramycin-acquired increasing rates of colonization resistance in the ICU by 1000 days and adjusted by the rate of resistances at admission were 0.82 (95% CI, 0.56 to 1.95; not statistically significant [NS]) and 1.13 (95% CI, 0.75 to 1.70; NS), respectively. The highest estimated rates of colistin- and tobramycin-resistant colonization by 1000 days in the ICU were 1.2 and 1.1 per 1000 days of ICU stay, respectively (Table 6). A summary of the study findings is shown in Fig. 2.

**Table 5:** Colonization in surveillance samples by colistin- and tobramycin-resistant pathogens

Variables	SDD period (between October 2011 and September 2015)				
	Total	1st year	2nd year	3rd year	4th year
	(*n* = 285)	(*n* = 59)	(*n* = 56)	(*n* = 69)	(*n* = 101)
Male sex, %	66.7	67.8	71.4	60.9	67.2
Age, years, mean ± SD	60.7 ± 15.0	56.2 ± 14.4	61.0 ± 16.0	61.3 ± 12.4	62.4 ± 16.1
Total patients	3948	1067	1069	851	961
Patients with SDD	1998	522	381	430	665
Colistin					
Resistance at ICU admission	113 (39.6)	5 (8.5)	17 (30.4)	30 (43.5)	61 (60.4)
Development of resistance	30 (10.5)	3 (5.1)	8 (14.3)	7 (10.1)	12 (11.9)
Observed (at ICU admission)					
Rate/100 patients	2.86	0.47	1.59	3.53	6.35
Rate/100 patients SDD	5.66	0.96	4.46	6.98	9.17
Estimated (acquired in ICU)					
Rate/100 patients	0.76	0.28	0.75	0.82	1.25
Rate/100 patients SDD	1.5	0.57	2.1	1.63	1.8
Tobramycin					
Resistance at ICU admission	151 (52.9)	17 (6.0)	32 (11.2)	34 (11.9)	68 (23.9)
Development of resistance	30 (10.5)	1 (0.4)	3 (1.1)	15 (5.3)	11 (3.9)
Observed (at ICU admission)					
Rate/100 patients	3.82	1.59	2.99	3.99	7.08
Rate/100 patients SDD	7.56	3.26	8.4	7.91	10.23
Estimated (acquired in ICU)	–				
Rate/100 patients	0.76	0.09	0.28	1.76	1.14
Rate/100 patients SDD	1.5	0.19	0.79	3.49	1.65

*ICU* Intensive care unit, *SDD* Selective digestive tract decontamination

**Table 6:** Evolution of rates of resistance to colistin and tobramycin in ICU, by 1000 days

	Resistance	Period			
		1st year	2nd year	3rd year	4th year
		(*n* = 59)	(*n* = 56)	(*n* = 69)	(*n* = 101)
Patient-days		9228	8583	10,731	9315
Colistin	At admission	5 (8.5)	17 (30.4)	30 (43.5)	61 (60.4)
	Acquired in ICU	3 (5.1)	8 (14.3)	7 (10.1)	12 (11.9)
	Acquired in ICU, by 1000 days	0.325	0.932	0.652	1.288
	Acquired in ICU, by 1000 days and adjusted by rate of resistance at admission^a^	0.278	0.228	0.187	0.153
Tobramycin	At admission	17 (6.0)	32 (11.2)	34 (11.9)	68 (23.9)
	Acquired in ICU	1 (0.4)	3 (1.1)	15 (5.3)	11 (3.9)
	Acquired in ICU, by 1000 days	0.108	0.350	1.398	1.181
	Acquired in ICU, by 1000 days and adjusted by rate of resistance at admission^a^	0.144	0.162	0.182	0.205

*ICU* Intensive care unit

The increasing rate of colistin- and tobramycin-acquired colonization resistance in the ICU by 1000 days and adjusted by the rate of resistance at admission was 0.82 (95% CI, 0.56 to 1.95; not statistically significant [NS]). *P* value for the goodness-of-fit test was 0.427. For tobramycin, the increasing rate was 1.13 (95% CI, 0.75 to 1.70; nonsignificant). *P* value for the goodness-of-fit test was 0.159

^a^Adjusted for values corresponding to first year, namely number of patients, number of resistances at admission, and exposure days

## Discussion

The main finding of the present study is a significant reduction in the incidence of infections caused by MDRB, including *Acinetobacter* spp., and other GNB- and ESBL-producing pathogens after 4 years of implementation of SDD in the daily care of ICU patients. Additionally, low rates of colistin- and tobramycin-resistant colonization were also observed in surveillance samples, with no significant increasing rate of ICU colonization resistance, by 1000 days, adjusted by the rate of resistances at ICU admission. In addition, VAP and secondary BSI infection rates declined. These findings were associated with a reduction in antibiotic consumption, which is a remarkable aspect of the present results.

We found a significant reduction of ICU-acquired infections caused by MDR-GNB following SDD in our ICU with a high level of antibiotic resistance before implementation of the SDD strategy. There is limited information on the effects of SDD in settings with high levels of MDRB. Four observational studies [7, 21–23] and one small randomized controlled trial [8] have been performed in ICUs where MDR-GNB were endemic or that had an outbreak of certain species of MDR-GNB. In these studies, SDD was applied either as a systematic treatment [21–23] or as a targeted approach for identified carriers [7, 8]. Most of these previous studies examined the effect of SDD on elimination or persistence of carriage of resistant strains, but ecological outcomes were not reported. Moreover, heterogeneity regarding settings and designs prevented clear interpretation of the findings; in fact, SDD was found to be useful in three studies [7, 21, 23] and failed in two of them [8, 22]. Brun-Buisson et al. [7] reported that SDD reduced colonization or carrier status and infection during an outbreak of ESBL-producing *Klebsiella pneumoniae*. Our study confirms that SDD can be useful in an environment with high levels of MDR-GNB.

However, the present results are in accord with data of studies carried out in settings with low levels of antibiotic resistance, including findings of systematic reviews of randomized controlled trials [3, 4] and long-term observational studies [24–27], confirming that SDD does not increase resistance. We also observed a significant reduction of infections caused by ESBL-producing MDRB. Similarly, Saidel-Odes and coworkers [28] reported that SDD decreased intestinal overgrowth of carbapenem-resistant *K. pneumoniae*. Zandstra et al. [29] also found that SDD is efficacious in controlling colonization with ESBL-producing bacteria, and Tascini et al. [30] showed that oral administration of gentamicin decontaminated the gastrointestinal tract and prevented infections caused by carbapenem-resistant *K. pneumoniae* strains producing *K. pneumoniae carbapenemase* (KPC)-type β-lactamase.

We also found a significant reduction of the incidence of infections caused by *Acinetobacter baumannii* and MDR-GNB. Similarly, in a randomized controlled study of 934 patients admitted to a surgical and medical ICU, of whom 466 were assigned to SDD and 468 to standard treatment (control subjects), colonization with gram-negative bacteria resistant to ceftazidime, ciprofloxacin, imipenem, polymyxin E, or tobramycin occurred in 16% of SDD patients and in 26% in the control group (*P* = 0.001) [31]. In a crossover study using cluster randomization in 13 ICUs in the Netherlands, the rate of isolation of gram-negative bacteria from rectal swabs was lower with SDD than with selective oropharyngeal decontamination (SOD) [4]. Also, SDD, as compared with standard care, was associated with a reduction of 57% of ICU-acquired bacteremia caused by glucose-nonfermenting gram-negative rods (*P. aeruginosa*, *Stenotrophomonas maltophilia*, and *Acinetobacter* spp.) and of 81% by *Enterobacteriaceae*, and these reductions were not accompanied by increases in intrinsic MDR-GNB colonization or infection [4]. A further analysis showed that development of ICU-acquired bacteremia caused by highly resistant microorganisms was 59% less frequent with SDD than with standard care and 63% less frequent with SDD than with SOD [32]. Recently, Camus et al. [33] found that the incidence rate of multidrug-resistant AGNB was lower during SDD (1.59 per 1000 patient-days versus preintervention 5.43%; *P* <  0.001) and also declined with time, concluding that a decontamination regimen did not favor the emergence of multidrug-resistant AGNB. In agreement with other studies, infections caused by *C. difficile* [31] and vancomycin-resistant *Enterococcu*s spp. [34] were not registered.

The use of SDD resulted in a significant reduction of VAP, which is consistent with previous observations. In a systematic review of randomized controlled trials of antibiotic prophylaxis in 6914 ICU patients collected from 36 trials, there was a significant reduction of respiratory tract infections in the treated group (OR, 0.28; 95% CI, 0.65 to 0.87) [3]. Also, in a study of 4945 mechanically ventilated patients admitted between 2005 and 2013, the incidence of VAP per 1000 ventilator days declined significantly from 4.38 ± 1.64 before to 1.64 ± 0.43 after introduction of SOD/SDD in December 2010 (*P* = 0.007) [35]. Implementation of SDD as the standard of care in ICUs is thus effective in preventing VAP.

A further remarkable finding of the study was a significant reduction of secondary BSI associated with the use of SDD. In a randomized study involving 16 Dutch ICUs, the proportion of ICU-acquired bacteremia by *Enterobacteriaceae* was lower for SDD than for SOD (OR, 0.38; 95% CI, 0.26 to 0.55; *P* <  0.001) [1]. In a systematic review of 51 randomized controlled trials conducted between 1987 and 2005, comprising 4079 patients treated with SDD and 3986 control subjects, SDD was associated with a reduction of overall and gram-negative BSIs of 27% and 61%, respectively, without affecting gram-positive BSIs [2]. Furthermore, prophylactic treatment with SDD was a protective factor for infections caused by MRDB. In a systematic review and meta-analysis of 64 studies assessing the effect of SDD and SOD on antimicrobial resistance, no differences were found in the prevalence of colonization or infection with gram-positive antimicrobial-resistant pathogens (MRSA, vancomycin-resistant enterococci) and gram-negative bacilli resistant to aminoglycosides and fluoroquinolones [36]. However, there was a reduction in polymyxin-resistant and third-generation cephalosporin-resistant gram-negative bacilli in recipients of SDD compared with those who did not receive the intervention. According to these data, the perceived risk of long-term harm related to SDD cannot be justified. The authors also conclude that the effect of SDD on ICU-level antimicrobial resistance rates is probably understudied. However, emergence of antimicrobial resistance is still a main objection to the widespread use of SDD in ICUs [5, 6, 8].

Also, there is a controversy regarding the emergence of an increased resistance to colistin and tobramycin used as part of SDD. We found low rates of colistin- and tobramycin-resistant colonization in cultures of surveillance samples during the 4-year SDD. It is known that there may be nosocomial transmission of highly resistant microorganisms from one patient infected to another, with or without SDD, and that this can increase the number of patients with GNB-resistant colonization [37]. As shown in Table 5, there are increases of colonization resistance to colistin and tobramycin at ICU admission. Also, the estimated rates adjusted to 100 patients with SDD decreased in the fourth year for tobramycin-resistant colonization and showed a small increase from 1.6 to 1.8 for colistin-resistant colonization in the third and fourth years of the study. The colistin- and tobramycin-acquired increasing rates of colonization resistance in the ICU by 1000 days and adjusted by the rate of resistances at admission were 0.82 (95% CI, 0.56 to 1.95; NS) and 1.13 (95% CI, 0.75 to 1.70; NS), respectively. These findings mean that although there were increases in the rates of colistin- and tobramycin-resistant colonization, these increases could not be associated with SDD and may have been linked to the progressive rise of MDR-GNB at ICU admission over the 4 years of the study and also may have been due to a higher degree of nosocomial transmission of highly resistant microorganisms among ICU patients. The highest estimated rates of colistin- and tobramycin-resistant colonization by 1000 days at risk were 1.2 and 1.1 per 1000 days, respectively (Table 6).

Colistin- and tobramycin-resistant colonization rates in our study were lower than 2.5/1000 patients days at risk, as shown in the study by Oostdijk et al. [38]. Using two large cohorts of ICU patients, Oostdijk et al. demonstrated that the prolonged use of colistin, as part of SDD and SOD, was not associated with increased acquisition of colistin-resistant GNB in the respiratory tract. Moreover, acquisition rates of colistin-resistant GNB in the intestinal tract during SDD ranged from 1.2 to 3.2 per 1000 patient-days at risk. The overall conversion rate from colistin susceptibility to resistance in the intestinal tract was below 1 conversion per 1000 patient-days at risk. During SDD, though, these conversion rates ranged from 3.2 to 5.4 per 1000 days of colonization with GNB and from 15.5 to 12.6 per 1000 days of colonization with tobramycin-resistant GNB. Also, the use of meropenem appeared to be strongly associated with the development of meropenem resistance in *P. aeruginosa* with an adjusted HR of 11.1 (95% CI, 2.4–51.5), corresponding to 23 events of resistance acquisition per 1000 patient-days at risk. [39]. On the basis of these findings, we concluded, as Oostdijk et al. [38] did, that the rates of resistance acquisition for frequently used antibiotics were considerably higher than for acquisition of colistin resistance during topical use of this agent.

Our findings differ from those of previous studies showing no increase in acquisition of resistant flora to these agents over a 5-year period [24] or no increases in the prevalence of resistance against colistin and tobramycin among gram-negative isolates during a mean of 7 years of SDD or SOD use [40]. Noteboom et al. [41] also observed that the percentages of antibiotic resistance with SDD and standard care were similar.

However, in a short course of SDD with colistin and gentamicin during an outbreak due to a KPC-2-producing *K. pneumoniae* strain, development of secondary resistance to colistin (19% increase in resistance rate) and gentamicin (45% increase) was found [8]. Halaby et al. [5] reported a significant relationship between use of SDD and tobramycin resistance as well as resistance to colistin among ESBL-producing pathogens. Brink et al. [6] showed the emergence of KPC in *Enterobacteriaceae* and the selection of strains resistant to colistin. Of note, Silvestri et al. [42], regarding data reported by Brink et al. [6], argued that an inadequate dose of enteral antimicrobials in the SDD protocol was responsible for the failure of *K. pneumoniae* to decolonize and eventually become resistant to colistin. Failure associated with subtherapeutic doses of SDD may cause overgrowth of MDR-GNB, with increased spontaneous mutation leading to polyclonality and resistance [43].

Associations between prolonged intravenous colistin use and development of colistin resistance have been reported from settings with high levels of carbapenemase-producing GNB [44, 45]. In contrast to facilitating resistance, SDD has been used successfully as a control measure in outbreak situations with ESBL-producing GNB [7, 46]. High intraluminal levels of topical antibiotics exceed minimum inhibitory concentrations of resistant pathogens, leading at least to temporary suppression, which reduces the risk of overgrowth and cross-transmission. However, there are several factors aside from SDD that produce GNB-resistant colonization. We did not find any MDR-GNB susceptible only to colistin in our study. Also, we observed decreased ICU global mortality over the course of the 4-year application of SDD.

Nevertheless, we think that SDD must be accompanied by careful monitoring of tobramycin and colistin resistance in GNB. We do so, as described in our protocol. We recommended screening weekly throughout the ICU stay.

## Conclusions

SDD in an ICU setting with a high level of resistance was associated with a clinically relevant reduction of infections caused by MDRB, with low rates of colistin- and tobramycin-resistant colonization and a nonsignificant increasing rate of ICU colonization resistance by 1000 days, adjusted by the rate of resistance at ICU admission. SDD was also a protective factor against MDRB infection. Furthermore, VAP and secondary BSI were significantly decreased after SDD. Notably, a decrease in antimicrobial consumption was also observed.

## Abbreviations

AGNB: Aerobic gram-negative bacilli
APACHE: Acute Physiology and Chronic Health Evaluation
BSI: Bloodstream infection
CLABSI: Central line-associated bloodstream infection
CLSI: Clinical and Laboratory Standards Institute
CT: Computed tomographic
CVC: Central venous catheter
ENVIN: National Nosocomial Infection Surveillance Study
ESBL: Extended-spectrum β-lactamase
EUCAST: European Committee on Antimicrobial Susceptibility Testing
GNB: Gram-negative bacilli
HELICS: Hospitals in Europe Link for Infection Control through Surveillance
ICU: Intensive care unit
KPC: *Klebsiella pneumoniae* carbapenemase
MDRB: Multidrug-resistant bacteria
MDR-GNB: Multidrug-resistant gram-negative bacilli
MRSA: Methicillin-resistant *Staphylococcus aureus*
MV: Mechanical ventilation
NS: Nonsignificant
RR: Risk ratio
SDD: Selective digestive tract decontamination
SEMICYUC: Spanish Society of Critical Care Medicine and Coronary Units
SOD: Selective oropharyngeal decontamination
VAP: Ventilator-associated pneumonia
WBC: White blood cells
